# Inverse association between Helicobacter pylori infection and childhood asthma in a physical examination population: a cross-sectional study in Chongqing, China

**DOI:** 10.1186/s12887-022-03682-8

**Published:** 2022-10-26

**Authors:** Donghai Wang, Yuxia Chen, Yuan Ding, Jinwei Tu

**Affiliations:** 1 Department of Respiratory Disease, Ministry of Education Key Laboratory of Child Development and Disorders, China International Science and Technology Cooperation Base of Child Development and Critical Disorders, Chongqing Key Laboratory of Pediatrics, National Clinical Research Center for Child Health and Disorders, Children’s Hospital of Chongqing Medical University, Chongqing, China; 2 Department of Gastroenterology, Ministry of Education Key Laboratory of Child Development and Disorders, China International Science and Technology Cooperation Base of Child Development and Critical Disorders, Chongqing Key Laboratory of Pediatrics, National Clinical Research Center for Child Health and Disorders, Children’s Hospital of Chongqing Medical University, Chongqing, China; 3 Department of Child Health Care, Ministry of Education Key Laboratory of Child Development and Disorders, China International Science and Technology Cooperation Base of Child Development and Critical Disorders, Chongqing Key Laboratory of Pediatrics, National Clinical Research Center for Child Health and Disorders, Children’s Hospital of Chongqing Medical University, Chongqing, China

**Keywords:** Asthma_1_, Children_2_, *Helicobacter pylori*_3_, Infecition_4_, Association_5_

## Abstract

**Background:**

Childhood asthma has substantial effects on children's health. It is important to identify factors in early life that influence childhood asthma. Accumulating evidence indicates that Helicobacter pylori may protect against allergic diseases. This study aimed to evaluate the relationship between H. pylori infection and pediatric asthma in Chongqing, China.

**Materials and methods:**

This cross-sectional study included healthy children aged 4–18 years who underwent a 13C urea breath test during medical checkups in 2021. All medical information was extracted from electronic medical records and a big data system. Logistic regression was used to evaluate the association between H. pylori infection and pediatric asthma, and multivariate regression models were adjusted for covariates.

**Results:**

In our study, 2241 participants, including 1240 boys (55.33%) and 1001 girls (44.67%), underwent urea breath testing (average age: 8.67 ± 2.70 years). Among them, 292 (13.03%) were positive for H. pylori and 152 (6.78%) had asthma. The rates of asthma diagnosis in H. pylori-negative and -positive children were 7.23% and 3.77%, respectively (odds ratio = 1.995; 95% confidence interval: 1.003–3.968; *P* < .05). Furthermore, family history of asthma and the percentage of eosinophils in routine blood examination were associated with childhood asthma; however, the body mass index, platelet count, and serum vitamin D level were not.

**Conclusions:**

We demonstrated a significant inverse association between H. pylori infection and pediatric asthma in Chongqing, China. Further studies are required to determine the causal association and underlying mechanisms to prevent and control childhood asthma.

## Introduction

Asthma is one of the most common chronic respiratory diseases worldwide and particularly affects children [1]. The latest data from the World Health Organization show that there are approximately 334 million patients with asthma worldwide [2], and approximately 250,000 deaths are attributable to asthma annually [3]. Several risk factors have been linked with asthma susceptibility, including host genetics, specific environmental exposures, and respiratory tract infections during early life. The classic risk factors include air pollution, smoking, infections, and personal allergy history, but the list is incomplete. Attention is turning to the role of the human microbiome in asthma pathogenesis and protection [4]. Identification of potential risk factors in cases of childhood asthma that are not explained by traditional risk factors might improve preventative strategies.

*Helicobacter pylori* is a spiral-shaped, Gram-negative, microaerophilic bacterium belonging to the genus Helicobacteraceae. It infects approximately 50% of the world population [5]. The prevalence of *H. pylori* infection varies widely between different parts of the world, with higher rates in developing countries. An up-to-date meta-analysis reported that the prevalence of *H. pylori* infection in China ranges from 35.4% to 66.4%, and varies according to socioeconomic status and the hygiene level [6]. In most instances, *H. pylori* colonizes the gastric stomach from early childhood [7]. *H. pylori* infection may lead to gastritis, peptic ulcer disease, gastric adenocarcinoma, and gastric mucosa-associated lymphoid tissue lymphoma; however, most infected subjects remain asymptomatic [8]. In addition, an increasing body of evidence indicates that there is a link between *H. pylori* infection and extra-gastric diseases [9].Recently, Sahin y et al. evaluated the association between neutrophil/lymphocyte ratio and mean platelet volume values with *H. pylori* infection, severity classification, and pre-and post-treatment status, however, no relationship was found [10].

As the incidence of asthma has increased, the prevalence of *H. pylori* infections has decreased. The hygiene hypothesis proposes that increased exposure to microorganisms in early life may confer a protective effect against asthma and allergic diseases [11]. Several epidemiological studies have reported an inverse association between *H. pylori* infection and allergic asthma, especially in children and adolescents [12, 13]. Nevertheless, There are still some conflicting views on whether there is an association between *H. pylori* infection and childhood asthma [14].

Our previous meta-analysis, which included 18 observational studies with 17,196 enrolled children, reported a significant negative association between *H. pylori* infection and the risk of childhood asthma, but lacked data about children from China [15]. Chongqing, a city in southwestern China, usually suffers from wet weather, and also most homes here have poor indoor ventilation, are usually plagued with dampness and mold, and prefer to use hygienic incense and mosquito coils, so that children in Chongqing have a significantly increased risk of childhood asthma [16]. Thus, we conducted a cross-sectional study to screen patients with childhood asthma and *H. pylori*-infected populations, to investigate the prevalence of childhood asthma and *H. pylori* infection in southwest China, and to explore the association between *H. pylori* infection and childhood asthma. Our findings can help to understand the different genetic and environmental contributors of asthma in order to better manage this disease.

## Methods

### Study population

This cross-sectional study included healthy children who underwent medical checkups at the Children’s Hospital of Chongqing Medical University from January 1, 2021 to December 31, 2021. Participants fulfilled all of the following inclusion criteria: 1) underwent a ^13^C urea breath test for *H. pylori* infection; 2) lived in urban areas of Chongqing; 3) had records of their sex, age, height, weight, family history of asthma, routine blood examination, and serum vitamin D test (not all children underwent a routine blood test and serum vitamin D test during physical examination); and 4) aged 4–18 years. The reliability of the ^13^C urea breath test results is lower for patients younger than 4 years due to technical difficulties performing the test in this age group; therefore, the analysis was limited to children aged 4–18 years. Participants who had a medical history of peptic ulcers or *H. pylori* infection were excluded. Participants who had received antibiotics, bismuth compounds, or proton pump inhibitors in the previous 2 weeks were also excluded. This study was exempted from informed consent and was approved by the Ethics Committee of the Children’s Hospital of Chongqing Medical University (approval number: K2022198). All procedures were performed in accordance with the guidelines of our institutional ethics committee and adhered to the tenets of the Declaration of Helsinki. All participants’ information was anonymous.

### Asthma diagnosis

Medical information of the children was retrieved from the electronic medical records and big data system of the hospital, including all diagnosis information and family history of various diseases. Asthma was defined as a diagnosis of “asthma” by a respiratory physician with lung function results in the big data system. This limitation was used to minimize the inadvertent inclusion of children who actually had alternative diagnoses such as bronchiolitis and viral-induced wheezing as infants that were misclassified by other pediatricians.

### Diagnosis of *H. pylori* infection

The ^13^C urea breath test is based on the ability of *H. pylori*-produced urease to hydrolyze swallowed urea labeled with ^13^C into carbon dioxide and ammonia. A baseline breath sample was collected by asking participants to exhale into a bag after overnight fasting. Then, a capsule containing ^13^C-urea was administered to the participants with 80–100 mL water. The second breath sample was obtained from participants after 30 min of sitting. *H. pylori* infection was determined by comparing the ^13^CO_2_ content of the baseline and 30 min samples, and a ratio ≥ 4.0 was considered positive (Shenzhen Zhonghe Headway Bio-Sci & Tech Co., Ltd, Guangdong, China). This was based on the Fourth Chinese National Consensus Report on the management of *H. pylori* infection [17].

### Other factors

The following factors were selected a priori and included in the analyses: sex, age, family history of asthma, body mass index (BMI), percentage of eosinophils in routine blood examination, and serum vitamin D level. Routine physical examinations, including measurement of height and weight, were performed by trained medical personnel. BMI was calculated by dividing weight in kilograms by height in meters squared to estimate general overweight and obesity. The BMI values corresponding to overweight and obesity differ according to age. The standards of overweight and obesity were based on the “Body mass index growth curves for Chinese children and adolescents aged 0 to 18 years” [18, 19]. Family history of asthma was reported based on first degree relatives (parents and siblings).

### Statistical analysis

Data for categorical variables(sex, family history of asthma, and *H. pylori* infection) are expressed as the number and percentage. Non-normally continuous distributed data(age, BMI, percentage of eosinophils in routine blood examination, and serum vitamin D level) are expressed as the median and interquartile range. Prior to investigating the association between *H. pylori* infection and asthma, univariate analyses were performed to identify possible confounders. The χ^2^ test and Mann–Whitney U test were used to compare the difference between the two groups. Logistic regression was used to test the association between *H. pylori* infection and asthma by calculating odds ratios (ORs) and 95% confidence intervals (CIs). Multivariate regression models were adjusted for covariates. Data were analyzed using Statistical Package for Social Sciences software (version 26.0). *P* < 0.05 was considered statistically significant.

## Results

### Characteristics of participants

**Fig. 1:**
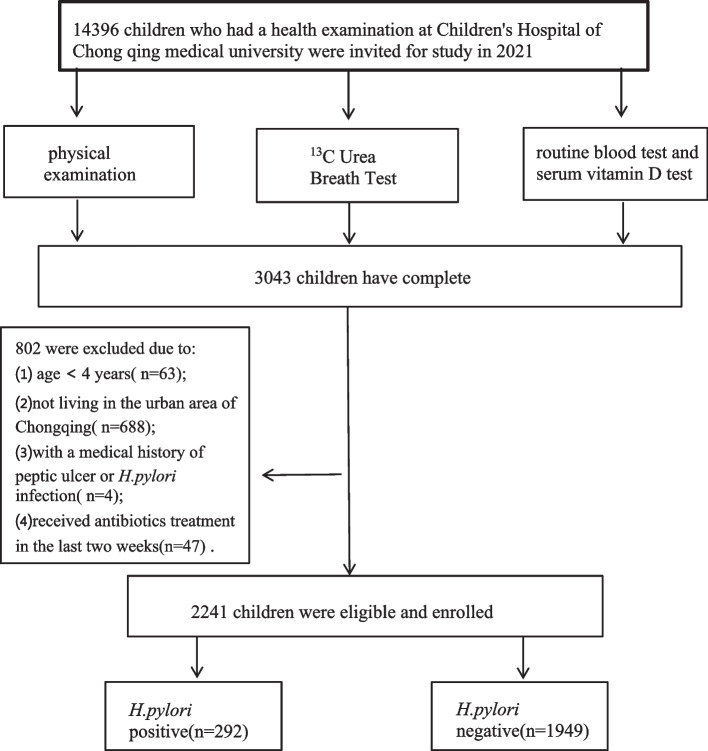
Flow chart of the study population enrollment

According to the inclusion and exclusion criteria, we selected 2241 participants aged 4–18 years (Fig. 1). The sample included 1240 boys (55.33%) and 1001 girls (44.67%) with a mean age of 8.67 ± 2.70 years. Among them, 292 (13.03%) participants were *H. pylori*-positive and 1949 (86.97%) participants were *H. pylori*-negative. The average number of outpatient visits for all participants was 21.45 ± 3.70. The number of outpatient visits did not significantly differ between the *H. pylori*-positive (22.50 ± 3.33) and *H. pylori*-negative (21.35 ± 2.92) groups (*P* > 0.05). A total of 152 (6.78%) participants were diagnosed with asthma. The number of outpatient visits was significantly higher for patients diagnosed with asthma (31.43 ± 5.58) than for patients not diagnosed with asthma (20.97 ± 3.74) (*P* < 0.05). Table 1 shows the basic characteristics of the subjects and univariate analysis of each factor related to asthma.

**Table 1:** Basic characteristics of the study participants and univariate analysis for each factor related to asthma

Characteristic	Total No. (%)	Asthma No. (%)	No asthma No. (%)	***P*** value	χ^2^/Z
Total	2241	152	2089		
Sex				.302	1.064
Male	1240 (55.33)	78(51.32)	1162(55.62)		
Female	1001(44.67)	74(48.68)	927(44.38)		
Age,years				.174	3.501
Median(P_25_,P_75_)	8.50(6.58,10.41)	8.21(6.25,9.73)	8.50(6.63,10.46)	.099	-1.648
4–5	395(17.63)	31(20.40)	364(17.43)		
6–11	1567(69.92)	109(71.71)	1458(69.79)		
12–18	279(12.45)	12(7.89)	267(12.78)		
BMI				.481	1.464
Median(P_25_,P_75_)	15.94(14.78,17.82)	15.95(14.81,17.75)	15.94(14.77,17.82)	.875	-0.158
No overweight	1802(80.41)	125(82.24)	1677(80.28)		
Overweight	317(14.15)	17(11.18)	300(14.36)		
Obesity	122(5.44)	10(6.58)	112(5.36)		
Family history				< .001	489.433
Positive	169(7.54)	81(53.29)	88(4.21)		
Negative	2072(92.46)	71(46.71)	2001(95.79)		
Platelet,10^12^					
Median(P_25_,P_75_)	285(244,327)	285(250,320)	285(243,327)	.869	-0.165
Vitamin D					
Median(P_25_,P_75_)	19.29(14.87,23.40)	19.52(14.84,23.69)	19.29(14.87,23.39)	.483	-0.702
Eosinophils(%)					
Median(P_25_,P_75_)	2.94(1.95,4.05)	4.01(2.05,5.96)	2.92(1.94,4.02)	< .001	-5.714
*H.pylori*				.028	4.829
Positive	292(13.03)	11(7.24)	281(13.45)		
Negative	1949(86.97)	141(92.76)	1808(86.55)		

*H.pylori* Helicobacter pylori, *BMI* Body mass index

### Association between *H. pylori* infection and asthma

There were 11 (3.77%) and 141 (7.23%) patients diagnosed with asthma in the H*. pylori*-positive and -negative groups, respectively, and this difference was significant (*P* < 0.05). The percentage of patients with a family history of asthma and the percentage of eosinophils in routine blood examination also significantly differed between the two groups (*P* < 0.05), but other characteristics (sex, age, BMI, platelet count, and serum vitamin D level) did not (*P* > 0.05). Univariate analysis revealed an association between *H. pylori* infection and asthma (*P* = 0.028), but the effects of other factors could not be ignored. A multinomial logistic regression model was further used to explore the factors that influenced asthma. We included all the factors in the basic multivariate logistic regression model, and this revealed an association between *H. pylori* infection and asthma (OR = 1.995, 95% CI: 1.003–3.968). Three adjusted multivariate logistic regression models were used to identify the potential association between *H. pylori* infection and asthma. There was an inverse relationship between *H. pylori* infection and asthma in all covariate-adjusted multivariate models (Table 2), suggesting that *H. pylori* infection is an independent protective factor for asthma (OR range: 1.887–2.008, *P* < 0.05).

**Table 2:** The risk of pediatric asthma according to the infection of *H.pylori*

Model	variant	OR(95%CI)	***P*** value
Basic model	*H.pylori*	1.995(1.003 ~ 3.968)	.049
	Family history	0.039(0.026 ~ 0.058)	< 0.001
	Eosinophils	1.079(1.022 ~ 1.139)	0.006
	Platelet	0.999(0.996 ~ 1.002)	0.511
	Vitamin D	0.997(0.967 ~ 1.027)	0.837
	BMI		0.422
	BMI(1)	1.005(0.452 ~ 2.234)	0.99
	BMI(2)	0.672(0.261 ~ 1.728)	0.409
	Age	1.006(0.931 ~ 1.086)	0.885
	Sex	0.758(0.517 ~ 1.112)	0.157
Model 1	*H.pylori*	1.887(1.007 ~ 3.538)	0.048
	Platelet	0.999(0.997 ~ 1.002)	0.582
	Vitamin D	1.008(0.982 ~ 1.035)	0.542
	BMI		0.577
	BMI(1)	0.783(0.397 ~ 1.545)	0.48
	BMI(2)	0.649(0.287 ~ 1.467)	0.299
	Age	0.948(0.887 ~ 1.013)	0.116
	Sex	0.854(0.611 ~ 1.193)	0.355
Model 2	*H.pylori*	1.960(1.040 ~ 3.692)	0.037
	Eosinophils	1.112(1.063 ~ 1.163)	< 0.001
	Platelet	0.999(0.997 ~ 1.002)	0.579
	Vitamin D	1.008(0.981 ~ 1.035)	0.579
	BMI		0.564
	BMI(1)	0.740(0.374 ~ 1.465)	0.388
	BMI(2)	0.641(0.283 ~ 1.451)	0.286
	Age	0.947(0.885 ~ 1.013)	0.111
	Sex	0.812(0.579 ~ 1.138)	0.227
Model 3	*H.pylori*	2.008(1.008 ~ 4.000)	0.047
	Family history history	0.036(0.024 ~ 0.054)	< 0.001
	Platelet	0.999(0.996 ~ 1.002)	0.551
	Vitamin D	0.997(0.967 ~ 1.027)	0.822
	BMI		0.393
	BMI(1)	1.049(0.473 ~ 2.327)	0.906
	BMI(2)	0.691(0.269 ~ 1.772)	0.441
	Age	1.009(0.935 ~ 1.089)	0.822
	Sex	0.787(0.538 ~ 1.152)	0.217

Model 1 is adjusted for family history and eosinophils. Model 2 is adjusted for family history. Model 3 is adjusted for eosinophils

*OR* Odd ratio, *CI* Confidence intervals

## Discussion

This study of 2241 children who underwent urea breath testing in 2021 shows that H. pylori infection is an independent predictor for a positive diagnosis of pediatric asthma. To the best of our knowledge, this is the largest study documenting such an association for children in China and the first to use pediatric urea breath test results rather than serologies, which are considered less reliable in children from a healthy childhood population.

The Children’s Hospital of Chongqing Medical University is a medical center for children in southwest China and the only children’s hospital in Chongqing. All the participants lived in urban areas of Chongqing, meaning that the effect of living in an urban area, which is an independent risk factor for pediatric asthma [20], was eliminated. The average number of outpatient visits was higher than 20, and almost all disease information was recorded by electronic medical records and a big data system of the hospital. It was considered more reliable to use asthma diagnoses recorded by a respiratory physician with lung function results in the system rather than questionnaires completed by parents. In addition, we included two asthma-related laboratory tests, the percentage of eosinophils in routine blood examination and the serum vitamin D level, which were not included in previous studies. Moreover, there was no heterogeneity in the study population, including differences in sex, age, BMI, and other confounders. To eliminate the possible influence of other variables, propensity score matching (PSM) analysis was performed using a logistic regression model based on age, sex, and other confounders to assess the relationship between H. pylori infection and childhood asthma. PSM refers to screening of the experimental and control groups using certain statistical methods so that the screened study subjects are comparable in terms of clinical characteristics (adjusting for potential confounders). However, there was little difference between matched and unmatched data; therefore, we did not describe PSM in Section Methods. This homogeneity may improve the comparability of our study to a certain extent.

The prevalence of childhood asthma in our study was 6.78%, which is higher than reported by epidemiological surveys in 2013 in Chongqing (4.43%) [21] and a meta-analysis in 2020 in China (2.6%) [22]. This may be because the prevalence of childhood asthma has increased in recent years and the prevalence of asthma is high in Chongqing [21], and all participants of our study lived in urban areas. The H. pylori infection rate was 13.03% in the present study. This is lower than that reported by Ren et al. in a meta-analysis, which demonstrated that the pooled prevalence of H. pylori infection was 44.2% in mainland China, 46.6% in Southwest China, and 28.0% in children and adolescents [6]. This difference may be because the prevalence of H. pylori infection increases with age and varies across geographic areas [5, 6]. In addition, all our participants lived in urban areas and had better sanitary conditions, higher family incomes, and healthier lifestyles.

Our study showed an inverse association between H. pylori infection and asthma in children, consistent with our previous meta-analysis [15]. Furthermore, several other studies have suggested that H. pylori infection significantly protects children younger than 10 years against allergic asthma [23, 24]. Zevit et al. demonstrated that the prevalence of asthma was 7.3% in children with H. pylori infection and 9.1% in healthy children, meaning that the prevalence of asthma was reduced in patients with H. pylori infection [12]. Another case–control study performed in Greece, which included 27 pediatric patients with asthma and 54 controls, also reported an inverse association between H. pylori infection and asthma [25]. Moreover, a cohort study performed in 2020 found that 16.4% of children who were H. pylori-negative at 2 and 10 years of age had asthma at 16 years of age, whereas children who were H. pylori-positive at 12 years of age did not have asthma at 16 years of age, suggesting that early exposure to H. pylori can prevent asthma [26]. The results of these studies are consistent with ours.

The possible protective mechanisms of *H.pylori* against allergic asthma, including (1) according to the “hygiene hypothesis”, infectious agents can inhibit allergic T helper (Th) 2 cell response, thus eliciting a Th1-type immune response, (2) *H.pylori* neutrophil-activating protein(HP-NAP) is one of the major virulence factors of *H. pylori*, which can increase interferon-γ production and decrease the level of interleukin-4, thereby stimulating Th1 activation and attenuating Th2 response in allergy-related asthma, (3)Another possibility involves the inverse correlation between *H. pylori* infection and gastroesophageal reflux disease (GERD), because gastroesophageal reflux can induce or aggravate asthma, but *H.pylori* can reduce gastroesophageal reflux, (4) *H. pylori* can also inhibit dendritic cells to promote immune tolerance and enhance the protective effect against asthma, and this inhibition is highly dependent on the suppressed regulatory T cells, (5) The gut-lung axis theory suggests remote regulation of lung immune function by *H. pylori,* (6)*H. pylori* infection may also prevent allergic asthma by altering stomach hormones levels, affecting the autonomic nervous system, and reducing the expression of heat shock protein 70 [27, 28].

It is well known that family history of allergies and blood eosinophil counts are strongly associated with childhood asthma [29, 30]. Recently, the association between BMI and childhood asthma has received increasing attention. Several studies have shown that BMI is significantly associated with the prevalence and incidence of allergic asthma [31, 32]. However, our study did not show such relationship. A recent systematic review and meta-analysis of case–control studies of children by Azizpour et al. [31] reported that the risk of asthma was 1.64 times and 1.92 times higher in individuals who were overweight and obese than in individuals who were underweight/normal weight, respectively. However, by removing the confounding factors, the effect size was reduced from 1.64 and 1.92 to 1.30. This suggests that although obesity increases the risk of asthma, but the confounding factors might influence the relationship between BMI and asthma. Therefore, in future epidemiological studies, it is important to investigate the confounding factors that affect the relationship between BMI and asthma. In addition, we found that children with asthma had similar vitamin D leves to healthy children, consistent with the previous studies by Yang et al. and Reinehr et al. [33, 34]. However, in contrast to our study, a meta-analysis by Wang et al., which included 35 studies with 27,271 participants, reported that children with asthma had significantly lower vitamin D levels than children without asthma. Furthermore, children with asthma who received vitamin D supplements had significantly lower recurrence rates than those who received a placebo [35].

Our study has several limitations. First, it was a single-center study and the results may not reflect the general population. Second, the participants only underwent urea breath testing, not endoscopy with biopsy, and there was a lack of data about the cytotoxin-associated gene A status, which is an important factor in H. pylori infection and is negatively associated with asthma and allergies [36]. Third, other potential confounding factors that might interfere with asthma, such as passive smoking, birth mode, and early antibiotic use, were not collected. Finally, our study was cross-sectional and lacked a comparison intervention, and its results only reflected epidemiological links, but failed to draw a cause-effect inference. Larger studies with an appropriate epidemiological design are required to investigate the potential causal relationship between H. pylori infection and asthma in the future.

## Conclusion

Our study demonstrated an inverse relationship between *H. pylori* infection and asthma in children. Further studies are required to determine the causal association and specific mechanisms, which will help to provide a scientific basis for the establishment of preventative and therapeutic strategies for childhood asthma.

## Abbreviations

BMI: Body mass index
CI: Confidence interval
GERD: Gastroesophageal reflux disease
OR: Odds ratio
PSM: Propensity score matching
Th: T helper
HP-NAP: *H.pylori* Neutrophil-activating protein
